# FHL1 protein isoforms in Emery-Dreifuss muscular dystrophy

**DOI:** 10.1186/1750-1172-10-S2-O18

**Published:** 2015-11-11

**Authors:** Esma Ziat, Anne T Bertrand

**Affiliations:** 1 Sorbonne Universités, UPMC Univ Paris 06, INSERM UMRS974, CNRS FRE3617, Center for Research in Myology, Paris, France

## 

Emery-Dreifuss muscular dystrophy (EDMD) is a hereditary muscular disorder characterized by early joint contractures, progressive muscular wasting and weakness of scapuloperoneal distribution, and at adult age, patients develop cardiac abnormalities with a high risk of sudden death[1]. EDMD encompasses both X-linked and autosomal inheritance due to mutations in the genes encoding the nuclear envelope proteins emerin, lamin A/C[2-4]. First mutations in the *Four-and-a-half LIM domain 1 gene* (FHL1) being responsible for X-linked EDMD were described by Gueneau et al.[5]. The human *FHL1* gene encodes three alternatively spliced isoforms, named FHL1A, FHL1B and FHL1C, with FHL1A being the most abundantly expressed protein isoform in striated muscle. There is still little known about the precise localization and functions of the three different FHL1 isoforms in human skeletal muscle. Here, we describe for the first time the subcellular localization of FHL1A, FHL1B, and FHL1C *in vitro* in differentiating human primary myoblasts.

Localization of FHL1 protein isoforms was studied at the myoblast and myotube stages by confocal microscope analysis. Endogenous FHL1B protein localization was detected by an anti-FHL1B specific antibody, while for FHL1A and FHL1C, as no efficient isoform-specific antibodies were available, an anti-Flag antibody was used to follow Flag-tagged FHL1A and Flag-tagged FHL1C protein expression, after lentiviral transduction of human primary myoblasts. Successful transduction was confirmed by western blotting of whole extracts from myoblasts and myotubes using an anti-Flag antibody. In human myoblasts, Flag-FHL1A and Flag-FHL1C showed both a cytoplasmic and a nuclear distribution, while the nuclear staining was more pronounced in Flag-FHL1C transduced myoblasts. Endogenous FHL1B protein gave a moderate cytoplasmic and a strong nuclear staining. During 6- and 12-days of human myoblast differentiation, localization of all three FHL1 protein isoforms shifted from the nucleus to the cytoplasm. In addition, all FHL1 protein isoforms were observed to co-localize with phalloidin-stained actin fibers. Collectively, these results indicate differentiation-related changes in expression and subcellular localization of the human FHL1 protein isoforms.
